# The Association among Urinary Lead and Cadmium, Serum Adiponectin, and Serum Apoptotic Microparticles in a Young Taiwanese Population

**DOI:** 10.3390/nu15214528

**Published:** 2023-10-25

**Authors:** Chien-Yu Lin, Chi-Kang Wang, Fung-Chang Sung, Ta-Chen Su

**Affiliations:** 1 Department of Internal Medicine, En Chu Kong Hospital, New Taipei City 237, Taiwan; 00724@km.eck.org.tw; 2 School of Medicine, Fu Jen Catholic University, New Taipei City 242, Taiwan; 3 Department of Environmental Engineering and Health, Yuanpei University of Medical Technology, Hsinchu 300, Taiwan; ckwang@mail.ypu.edu.tw; 4 Department of Health Services Administration, China Medical University College of Public Health, Taichung 404, Taiwan; fcsung@mail.cmu.edu.tw; 5 Department of Food Nutrition and Health Biotechnology, Asia University, Taichung 413, Taiwan; 6 Department of Environmental and Occupational Medicine, National Taiwan University Hospital, Taipei 100, Taiwan; 7 Department of Internal Medicine and Cardiovascular Center, National Taiwan University Hospital, Taipei 100, Taiwan; 8 Institute of Environmental and Occupational Health Sciences, College of Public Health, National Taiwan University, Taipei 100, Taiwan; 9 The Experimental Forest, College of Bio-Resources and Agriculture, National Taiwan University, Nantou 558, Taiwan

**Keywords:** adiponectin, adolescent, apoptotic microparticles, atherosclerosis, cadmium (Cd), environmental health hazards, lead (Pb), programmed cell death, Taiwan

## Abstract

Previous studies reported that lead (Pb) and cadmium (Cd) exposure are linked to changes in serum adiponectin; an adipokine that promotes glycolysis and inhibits gluconeogenesis to regulate glucose metabolism. However, no study has ever explored the relationship between exposure to these two heavy metals and adiponectin in adolescents and young adults. Additionally, the role of adiponectin in the relationship between Pb and Cd exposure and vascular endothelial cell apoptosis has never been investigated. In this study, 724 Taiwanese participants, aged 12 to 30 years, were enrolled to investigate the association among urinary lead and cadmium, serum adiponectin, and apoptotic microparticles (CD31+/CD42a−, CD31+/CD42a+, and CD14). The results of the current study revealed a statistically significant inverse association between urine Pb and Cd levels and adiponectin levels, as well as a positive association with apoptotic microparticles (CD31+/CD42a−, CD31+/CD42a+, and CD14). Adiponectin was also inversely correlated with CD31+/CD42a− and CD31+/CD42a+. Moreover, when subjects with both Pb and Cd levels above the 50th percentile were compared to those below it, the former group exhibited the lowest average adiponectin value. Additionally, a more pronounced positive association between heavy metals and apoptotic microparticles (CD31+/CD42a− and CD31+/CD42a+) was observed when adiponectin levels were lower. Furthermore, an interaction between adiponectin and heavy metals was identified in the relationship between these metals and CD31+/CD42a−. In conclusion, these findings suggest that Pb and Cd exposure may have an adverse effect on adiponectin, and it may play a role in the link between heavy metal exposure and the dysfunction of vascular endothelial cells. Future studies are needed to establish whether a causal relationship exists.

## 1. Introduction

Heavy metal pollution from industrial activities, agriculture, waste disposal, combustion processes, and natural weathering poses environmental and health risks [1]. Evaluating the health effects of lead (Pb) and cadmium (Cd) exposure is crucial due to the widespread exposure to these heavy metals and their established health impacts [1]. These two toxic substances rank among the top ten chemicals that raise public health concerns, as stated by the World Health Organization [1]. Exposure to Pb and Cd increases oxidative stress and can damage lipids, proteins, and DNA, causing dangerous effects on various organ systems [2]. Despite efforts to decrease human exposure, low levels of Pb and Cd remain linked to adverse health outcomes in recent studies [3,4].

Cardiovascular diseases (CVD) are the primary reason for human mortality, despite substantial advancements in treatment [5]. In addition to conventional risk factors, such as diabetes mellitus, hypertension, and tobacco use, the impact of environmental Pb and Cd exposure on CVD has emerged as a critical concern in recent research [6,7,8]. Endothelial cell apoptosis inhibits nitric oxide production and promotes a pro-thrombotic and pro-inflammatory arterial environment. These mechanisms lead to monocyte and lipid accumulation, plaque destabilization, and impaired vascular endothelial function. This process plays a central role in the progression of atherosclerosis [9,10]. Moreover, apoptosis leads to the generation of apoptotic microparticles comprising a variety of bioactive molecules that initiate a pro-inflammatory response [11]. These microparticles can disrupt the normal functioning of healthy endothelial cells, leading to reduced nitric oxide production, inflammation, and oxidative stress. As such, apoptotic microparticles are widely recognized as indicators of vascular endothelial cell dysfunction in the context of atherosclerosis [11]. In our previous study, we conducted a cohort study involving adolescents and young adults in Taiwan, uncovering a positive correlation between urinary levels of Pb and Cd and serum apoptotic microparticle levels [12]. The findings of this study imply that these two heavy metals could potentially disrupt vascular endothelial cell functions in this young population [12].

Adiponectin, an adipokine synthesized by adipocytes, exerts regulatory functions on many metabolic pathways [13,14]. Notably, it augments insulin sensitivity, thus mitigating the risk of diabetes. Moreover, adiponectin exerts anti-inflammatory effects, preserves endothelial function, impedes atherosclerotic progression, and demonstrates anti-cancer properties, encompassing the inhibition of cancer cell proliferation and the modulation of immune responses [13,14]. Investigations have brought to light the significance of adiponectin in enhancing the function of vascular endothelial cells. Adiponectin plays a vital role in the intricate communication pathways within endothelial cells and has the ability to protect against abnormal vascular remodeling by inhibiting the growth and migration of smooth muscle cells [15]. Moreover, experimental research has shown that adiponectin has the potential to reduce microparticle quantities in both laboratory settings and living organisms [16]. These findings align with our earlier discussion of adiponectin’s protective effects on vascular endothelial cell function, suggesting that it may play a role in mitigating the detrimental effects of microparticles on vascular health. Furthermore, clinical trials have provided additional support for this concept, demonstrating a negative association between the serum levels of adiponectin and the microparticle levels in patients with diabetes and hyperlipidemia [17,18]. These collective findings reinforce the potential value of adiponectin in addressing vascular endothelial dysfunction and its associated complications.

Previous experimental studies have consistently indicated that exposure to high doses of Pb or Cd can adversely impact adiponectin levels [19,20]. Moreover, an occupational study involving welders exposed to heavy metals, including Pb and Cd, also reported lower levels of serum adiponectin [21]. However, research on the association between Pb or Cd exposure and adiponectin levels in the general population remains limited and inconclusive. Some mother–infant studies have found no significant association [22,23]. Conversely, in an elderly population, a study reported a positive association [24], while in another cohort study involving women, a negative association was discovered [25]. Despite these existing studies, there is a notable gap in the literature, as no previous investigations have explored this association in adolescents and young adults.

In summary, prior investigations have demonstrated that exposure to Pb or Cd is linked to CVD and the dysfunction of endothelial cells. The existing literature also indicates that these two heavy metals may detrimentally affect adiponectin levels, and higher adiponectin levels have been associated with improved endothelial function. However, there is a lack of specific research exploring the role of adiponectin in the relationship between heavy metal exposure and vascular endothelial function. Moreover, the association between these heavy metals and adiponectin remains unexplored in adolescents and young adults. Additionally, considering that humans are often exposed to both Pb and Cd simultaneously, it would be valuable to investigate the combined effects of co-exposure on adiponectin and vascular endothelial function. To address this research gap comprehensively, our study aims to provide valuable insights into the potential association between Pb or Cd exposure and adiponectin levels in this particular age group. In this Young Taiwanese Cohort (YOTA) study, we measured urine levels of Pb and Cd, as well as serum adiponectin, and apoptotic microparticles (CD31+/CD42a−, CD31+/CD42a+, and CD14).

## 2. Materials and Methods

### 2.1. Study Population

**Figure 1:**
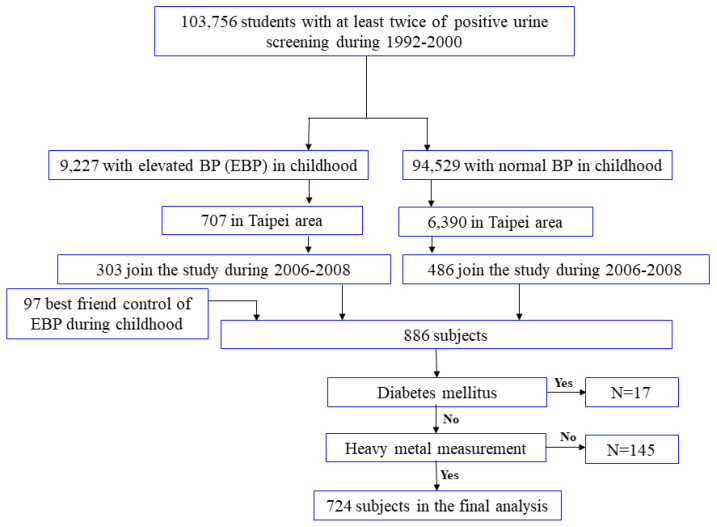
Flow chart detailing the participant selection procedure.

During the period of 2006 to 2008, we established the YOTA cohort by enrolling 886 individuals aged between 12 and 30 years, based on their childhood blood pressure levels, whether elevated or normal [26]. The research protocol for this study was approved by the Research Ethics Committee of the National Taiwan University Hospital (NTUH-9561705054), and all participants provided signed consent forms. To ensure the integrity of our study, we excluded 17 individuals with diabetes, as their medication could potentially influence adiponectin levels [27]. Additionally, 145 participants were excluded due to incomplete data on urine heavy metal levels. Consequently, our final analysis included a total of 724 participants. A flow chart of this study is shown in Figure 1. For more comprehensive information, please refer to the Supplementary section.

### 2.2. Measurement of Serum Adiponectin, Urinary Pb and Cd Levels

Following an overnight fast, blood samples were collected from all participants, and the levels of adiponectin in the serum were determined using a commercially available immunoassay kit (R&D Systems, Minneapolis, MN, USA). The comprehensive methodology for measuring urinary levels of Pb and Cd in this study has been thoroughly described and can be found in our previously published articles [28,29]. The lowest detectable concentrations for Pb and Cd were 0.007 μg/L and 0.006 μg/L, respectively. Further details can be accessed in the Supplementary Materials.

### 2.3. Measurement of the Concentration of Apoptotic Microparticles in Serum

The origin of apoptotic microparticles can be determined through the use of specific markers, such as CD31 and CD42a. While CD31 is present on both apoptotic platelets and endothelial cells, CD42a is exclusively found on apoptotic platelets. Thus, the presence of CD31+ and the absence of CD42a (CD31+/CD42a−) indicate that the microparticles originate from apoptotic endothelial cells. On the other hand, the presence of both markers (CD31+/CD42a+) suggests that they derive from apoptotic platelets [30,31]. Additionally, CD14, another marker expressed on monocytes, macrophages, and neutrophils, plays a significant role in the phagocytosis of apoptotic cells by macrophages [32]. To measure these three types of apoptotic microparticles, we utilized flow cytometry, following a previously established method [33]. In brief, citrated serum was subjected to analysis using fluorescent monoclonal antibodies: phycoerythrin-labeled anti-CD31, anti-CD42a, and anti-CD14 (BD Bioscience). The levels of microparticles are expressed as counts per microliter (counts/µL) [33].

### 2.4. Covariates

This study incorporated various covariates in its analysis, encompassing age, sex, household income, hypertension, smoking status, alcohol consumption, and z score of body mass index (BMI). Further elaboration on these covariates can be found in the Supplementary section.

### 2.5. Statistical Analysis

Statistical analyses were performed using SPSS for Windows (version 20.0; SPSS Inc., Chicago, IL, USA). In this research, urinary creatinine served as an adjustment for urine flow since the concentrations of heavy metals in urine can be affected by the flow rate [34]. Variables showing notable deviations from a normal distribution underwent a natural logarithm transformation prior to further analyses. Multiple linear regression models were conducted to assess the association of various factors with the variables of interest. Specifically, we investigated the regression coefficients of natural logarithm-transformed adiponectin and apoptotic microparticles, accounting for the influence of logarithm-transformed heavy metals and adiponectin within the regression model. Our analysis encompassed a comprehensive set of covariates, including age, sex, BMI z score, smoking habits, alcohol consumption, and household income. Moreover, we evaluated the geometric means (with standard error) of serum adiponectin across different quartiles of urinary heavy metals and in separate subgroups of Pb and Cd using multiple linear regression models. Additionally, we examined the regression coefficients of logarithm-transformed apoptotic microparticles with a one-unit increase in logarithm-transformed heavy metals for varying levels of adiponectin, specifically focusing on the 50th percentile. We conducted an examination of interaction tests by incorporating cross-product terms into multiple linear regression models. A significance level of *p* < 0.05 was considered statistically significant in our analyses.

## 3. Results

Out of the 724 participants included in this study, 434 were women, while 290 were men, and the average age (SD) was 21.2 (3.4) years. The detection rates for both Pb and Cd were found to be 100%. Pb and Cd exhibited a strong positive association, as indicated by a Spearman’s correlation coefficient of 0.815 (*p* < 0.001). Out of the entire pool of participants, data on adiponectin were only available for 362 individuals. Detailed demographic information, along with the measurements of urinary heavy metals, serum adiponectin, and apoptotic microparticles, can be found in Table 1 and Table 2. The geometric means (95% confidence interval) of the urinary heavy metal concentrations across various categories are presented in Supplementary Table S1. Notably, levels of Pb and Cd were significantly elevated in the younger population (12–19 years old) (*p* < 0.001) and among women (*p* < 0.001). Furthermore, urinary Pb levels were higher among subjects with hypertension (*p* = 0.030), while Cd levels were lower among active smokers (*p* = 0.011). Table 3 illustrates the relationships observed among these heavy metals, adiponectin, and apoptotic microparticles. We observed that the levels of urinary Pb and Cd exhibited a negative association with adiponectin concentrations, while displaying a positive association with CD31+/CD42a−, CD31+/CD42a+, and CD14 counts. Additionally, the levels of adiponectin were found to be negatively correlated with CD31+/CD42a− and CD31+/CD42a+.

Table 4 presents the geometric mean of serum adiponectin in relation to different quartiles of urinary heavy metals, as analyzed through multiple linear regression models. Notably, a significant decline in the mean levels of adiponectin was evident with increasing quartiles of Pb and Cd (*p* for trend < 0.001). As a further insight, both Pb and Cd at the highest quartile were substantially lower when compared to the lowest quartile, with both exhibiting a *p* value < 0.001. Table 5 illustrates the geometric mean (with standard error) of adiponectin within distinct subgroups of Pb and Cd (cut at the 50th percentile). These results indicate a significant reduction in adiponectin levels as the concentrations of Pb and Cd subgroups increase (*p* for trend < 0.001). Comparing subjects with Pb and Cd levels below the 50th percentile to those above the 50th percentile, the latter group had the lower mean adiponectin value (geometric mean (SE) = 3987.82 (1.13) ng/mL; *p* < 0.001). Moreover, Supplementary Table S2 reveals a noteworthy elevation in adiponectin levels with increasing concentrations of Pb and Cd subgroups (all *p* for trend < 0.05). Notably, subjects with Pb and Cd levels above the 50th percentile displayed the highest mean values for CD31+/CD42a−, CD31+/CD42a+, and CD14 counts, in comparison to subjects with Pb and Cd levels below the 50th percentile.

**Figure 2:**
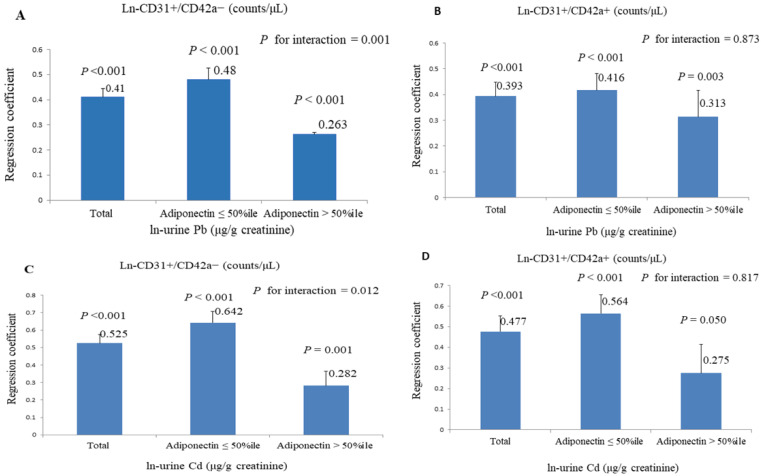
The regression coefficients (SE) of natural-logarithm-transformed serum apoptotic microparticles concerning an increase in the natural logarithm-transformed urine concentrations of Pb and Cd at different adiponectin levels (cut at the 50th percentile). (**A**) Pb and CD31+/CD42a−. (**B**) Pb and CD31+/CD42a+. (**C**) Cd and CD31+/CD42a−. (**D**) Cd and CD31+/CD42a+.

Figure 2 displays the regression coefficients of natural-logarithm-transformed serum apoptotic microparticles concerning an increase in the natural-logarithm-transformed urine concentrations of Pb and Cd. Our study also examined different concentrations of adiponectin and the interplay between adiponectin and these two heavy metals, with the concentrations divided at the 50th percentile. Subjects with adiponectin levels above the 50th percentile exhibited lower regression coefficients compared to those below it. Furthermore, the levels of Pb and Cd demonstrated positive associations with CD31+/CD42a− and CD31+/CD42a+, except for the relationship between Cd and CD31+/CD42a+ in individuals with adiponectin levels above the 50th percentile. In the association between the heavy metals and CD31+/CD42a−, adiponectin levels interacted with these two elements (*p* for interaction was found to be 0.001 for Pb and 0.012 for Cd, respectively), whereas no interaction was observed between adiponectin and the heavy metals concerning CD31+/CD42a+.

## 4. Discussion

The results of this study revealed a statistically significant inverse association between urinary Pb and Cd levels and adiponectin levels, as well as a positive association with apoptotic microparticles (CD31+/CD42a−, CD31+/CD42a+, and CD14). Adiponectin was also inversely correlated with CD31+/CD42a− and CD31+/CD42a+. Furthermore, the positive association between heavy metals and apoptotic microparticles (CD31+/CD42a− and CD31+/CD42a+) was more pronounced when adiponectin levels were lower, and an interaction between adiponectin and heavy metals was identified in the association between the two metals and CD31+/CD42a−. These results indicate that decreased adiponectin might play a role in the connection between heavy metal exposure and dysfunction of vascular endothelial cells. The importance of this research is threefold. First, investigating the health effects of heavy metal exposure in adolescents and young adults is an important area of investigation, as this age group is less likely to have medical diseases that could potentially complicate these associations. Second, this study is innovative in its exploration of the complex relationships between heavy metal exposure, adiponectin, and apoptotic microparticles. Moreover, this is the first study to investigate the association between heavy metals and adiponectin in adolescents and young adults. Finally, if the associations identified in this study are found to be causal, the prevention of heavy metal exposure would be an important public health strategy.

Adiponectin actively contributes to the prevention of atherosclerosis in both the endothelial cells and the subendothelial space [15]. Adiponectin exerts atheroprotective effects by increasing nitric oxide production, reducing platelet aggregation, and inhibiting the transformation of monocyte-derived macrophages into foam cells [35,36,37]. Additionally, adiponectin has also been demonstrated to have anti-proliferative and anti-migratory properties in smooth muscle cells [38]. In an experimental study, adiponectin limited the release of microparticles from lipopolysaccharide-treated monocytes [16]. Clinical investigations have also shown an inverse relationship between adiponectin levels and circulating endothelium–platelet microparticles. In a study of 191 hyperlipidemic patients with type 2 diabetes, Nomura et al. (2009) discovered that combined statin and omega-3 fatty acid treatment significantly reduced platelet-derived microparticles and increased in adiponectin levels [17]. Similarly, a study by Esposito et al. (2011) found that thiazolidinedione treatment decreased circulating endothelial microparticles and increased serum adiponectin levels in 110 patients with newly diagnosed type 2 diabetes [18]. In the current investigation, we observed an inverse association between serum adiponectin levels and CD31+/CD42a− and CD31+/CD42a+, consistent with previous experimental and clinical studies.

There is limited experimental research on the influence of Cd or Pb exposure on adiponectin regulation. Nevertheless, studies have indicated that Cd can decrease both adipocyte size and adiponectin secretion in mice [20]. Additionally, Pb exposure may also affect central adipokine signaling by decreasing the expression of adiponectin receptor 1b in the brain of zebrafish. However, the underlying mechanism is not yet understood [19]. Epidemiologic studies have investigated the relationship between Pb or Cd and serum adiponectin. An occupational study of 100 welding workers in Taiwan found that those with higher urinary levels of heavy metals, including Pb and Cd, had lower levels of serum adiponectin, suggesting that Pb exposure may be the main contributor to this association [21]. However, studies on the general population have yielded mixed results. A study of 1363 mother–infant pairs in Canada reported no significant association between maternal blood levels of Pb and Cd and umbilical cord blood adiponectin levels [22]. Similarly, a study of 411 mother–infant pairs in the U.S. found no association between maternal blood levels of these two heavy metals and serum adiponectin in children aged 4–6 years [23]. Conversely, a separate investigation involving 70 Canadian participants aged 46–87 years demonstrated a positive association between urinary Cd and serum adiponectin levels. Additionally, path analyses supported the notion that adiponectin acts as a mediator in the association between urinary Cd and urinary retinol binding protein, a biomarker indicative of renal dysfunction [24]. Another study evaluated 1228 women in the U.S., and found that urinary Cd, but not Pb, was associated with lower levels of adiponectin [25]. In our study, which enrolled a young Taiwanese population, we found that both urinary Pb and Cd concentrations were inversely related to serum adiponectin levels. We propose that differences between our results and those of previous studies may be due to variations in the demographic characteristics of the study participants, the size of the study groups, the types of samples collected, and the variables taken into account.

Atherosclerosis represents a multifactorial pathological process characterized by the accumulation of lipids, inflammation, and impaired endothelial function [39]. Evidence suggests that exposure to Pb and Cd can perturb lipid metabolism and initiate amyloid angiopathy, potentially contributing to the pathogenesis of atherosclerosis [40]. Additionally, the escalation of oxidative stress may induce detrimental effects on proteins and DNA, further compromising the integrity of vascular endothelial cells [2]. In a prior investigation on the same study cohort, a positive association was established between elevated levels of urine Pb and Cd and apoptotic microparticles, namely, CD31+/CD42a−, CD31+/CD42a+, and CD14 counts, indicative of vascular endothelial cell dysfunction [12]. Nevertheless, no epidemiologic study has hitherto explored the role of adiponectin in relation to the interplay between heavy metals and vascular endothelial cell dysfunction. In this present paper, we present the initial evidence suggesting that adiponectin could potentially act as a mediator in the association between exposure to Pb or Cd and endothelial cell dysfunction. Moreover, simultaneous exposure to low doses of Pb and Cd may lead to cumulative cellular damage through shared molecular pathways. Previous animal studies and epidemiological research have proposed that these metals may exert synergistic effects on diverse health indicators, including lipid metabolism, biomarkers of oxidative stress, renal function, and blood pressure [3,41,42,43]. In our prior investigation, we also found that the combined exposure to Pb and Cd might result in synergistic impacts on apoptotic microparticles [12]. Furthermore, our current study presents an initial indication of a plausible synergistic influence of low levels of Cd and Pb on serum adiponectin concentrations.

Our study has certain limitations that deserve consideration. Firstly, the cross-sectional nature of the study precludes us from drawing any causal inferences. Secondly, the study population was confined to a specific young ethnic group in Taiwan; thus, the generalizability of the conclusions to broader populations remains uncertain. Thirdly, our analysis did not encompass other environmental contaminants that could potentially influence both adiponectin and apoptotic microparticles, leaving room for the possibility that our findings are merely indicative of secondary associations.

## 5. Conclusions

In our comprehensive study of a cohort of young Taiwanese individuals, we made notable discoveries. Specifically, we observed a significant and remarkable association between elevated urinary levels of both Pb and Cd and a concomitant decrease in serum adiponectin levels, along with an increase in serum apoptotic microparticles. Interestingly, our investigation revealed that the effects of these two heavy metals on adiponectin are synergistic. Furthermore, our research revealed intricate interactions between these heavy metals, adiponectin, and apoptotic microparticles, underscoring the complex interplay among these variables. Taken together, these findings suggest that reduced adiponectin levels may play a pivotal role in mediating the apoptosis-related damage to vascular endothelial cells induced by exposure to Pb and Cd. This, in turn, provides a new perspective on the mechanistic basis of how Pb and Cd cause damage to endothelial cells and underscores the need for further in-depth investigations to definitively establish any causal relationships.

## Figures and Tables

**Table 1:** Demographic data in the studied Taiwanese population (categorical variables) (*n* = 724).

	Numbers (%)
Age (years): 12–19	231 (31.9)
20–30	493 (68.1)
Sex: Male	290 (40.1)
Female	434 (59.9)
BMI: <24	570 (78.7)
≥24	154 (21.3)
Smoking: Active	122 (16.9)
Not active	602 (83.1)
Drinking: Current	69 (9.5)
Not current	654 (90.5)
Household income: <TWD 50,000	282 (39.1)
≥TWD 50,000	440 (60.9)
Hypertension: Yes	54 (7.5)
No	670 (92.5)

Abbreviation: BMI: body mass index.

**Table 2:** Mean and geometric mean of adiponectin, urinary heavy metals, and apoptotic microparticles in the studied Taiwanese population (*n* = 724).

	Mean (SD)	Geometric Mean (Geometric SD)
Adiponectin (ng/mL) ^a^	14,401.22 (40,206.03)	6404.14 (2.99)
Urinary heavy metals		
Pb (μg/g creatinine)	6.98 (16.57)	1.47 (5.27)
Pb (μg/L)	9.22 (17.53)	2.18 (5.17)
Cd (μg/g creatinine)	1.43 (2.64)	0.62 (3.51)
Cd (μg/L)	2.00 (2.73)	0.92 (3.63)
Apoptotic microparticles (counts/µL)		
CD31+/CD42a−	243.73 (367.80)	166.47 (3.46)
CD31+/CD42a+	11,432.68 (18,428.00)	3725.29 (5.46)
CD14	123.46 (74.35)	108.03 (1.67)

^a^ *n* = 362. Abbreviations: Cd: Cadmium; Pb: Lead.

**Table 3:** Linear regression coefficients (standard error) of ln adiponectin and ln microparticles with a unit increase in ln heavy metals and ln adiponectin in multiple linear regression models.

	Ln Adiponectin	Ln CD31+/CD42a−	Ln CD31+/CD42a+	Ln CD14
	(ng/mL)	(counts/µL)	(counts/µL)	(counts/µL)
	*n* = 362	*n* = 722	*n* = 722	*n* = 722
Ln adiponectin (ng/mL)		−0.186 (0.064)	−0.277 (0.090)	−0.020 (0.027)
*p* value		0.004 *	0.002 *	0.465 *
Ln Pb (μg/g creatinine)	−0.169 (0.032)	0.349 (0.025)	0.320 (0.038)	0.067 (0.012)
*p* value	<0.001 *	<0.001 *	<0.001 *	<0.001 *
Ln Cd (μg/g creatinine)	−0.192 (0.045)	0.433 (0.033)	0.417 (0.050)	0.071 (0.015)
*p* value	<0.001 *	<0.001 *	<0.001 *	<0.001 *

Model adjusted for age, sex, BMI z score, smoking status, drinking status, household income. Abbreviations: Cd: cadmium; Pb: lead. * *p* < 0.05.

**Table 4:** Geometric means (standard error) of serum adiponectin at different quartiles of urinary heavy metals in multiple linear regression models (*n* = 362).

		Adiponectin (ng/mL)		
Heavy Metals (μg/g Creatinine)		Geometric Mean (SE)	*p*	*p* for Trend
Pb	≤0.36 (≤25%ile)	7065.65 (1.15)	Reference	<0.001
	≤0.61 (≤50%ile)	8014.44 (1.14)	1.000	
	≤2.00 (≤75%ile)	8160.00 (1.14)	1.000	
	>2.00 (>75%ile)	3725.66 (1.14)	<0.001	
Cd	≤0.20 (≤25%ile)	8184.52 (1.14)	Reference	<0.001
	≤0.32 (≤50%ile)	6967.42 (1.14)	1.000	
	≤0.75 (≤75%ile)	7608.34 (1.14)	1.000	
	>0.75 (>75%ile)	4101.06 (1,14)	<0.001	

Model adjusted for age, sex, BMI z score, smoking status, drinking status, household income. Abbreviations: Cd: cadmium; Pb: lead.

**Table 5:** Geometric mean (SE) of adiponectin in different heavy metal subgroups (cut at the 50th percentile) in the multiple linear regression model.

		Pb ≤ 50%ile and Cd ≤ 50%ile	Pb > 50%ile and Cd ≤ 50%ile	Pb ≤ 50%ile and Cd > 50%ile	Pb > 50%ile and Cd > 50%ile
	Number	191	34	34	103
Adiponectin (ng/mL)	Geometric mean (S.E.)	7427.91 (1.11)	8586.96 (1.21)	10,107.17 (1.21)	3987.82 (1.13)
	*p* value	Reference	0.442	0.101	<0.001
	*p* for trend	<0.001			

Model adjusted for age, sex, BMI z score, smoking status, drinking status, household income. Abbreviations: BMI: body mass index; Cd: cadmium; Pb: lead.

## Data Availability

The datasets produced and examined in the course of this study can be obtained upon reasonable request from the corresponding author.

## Supplementary Materials

The following supporting information can be downloaded at: https://www.mdpi.com/article/10.3390/nu15214528/s1. File S1: Geometric mean (SE) of apoptotic microparticles in different Pb and Cd subgroups in the linear regression model are described in the Supplementary Material file.

## References

[1] 10 Chemicals of Public Health Concern. https://www.who.int/news-room/photo-story/photo-story-detail/10-chemicals-of-public-health-concern.

[2] Fu Z., Xi S. (2020). The effects of heavy metals on human metabolism. Toxicol. Mech. Methods.

[3] Chen X., Zhu G., Wang Z., Zhou H., He P., Liu Y., Jin T. (2019). The association between lead and cadmium co-exposure and renal dysfunction. Ecotoxicol. Environ. Saf..

[4] Zhang H., Yan J., Niu J., Wang H., Li X. (2022). Association between cadmium and lead co-exposure, blood pressure, and hypertension: A cross-sectional study from northwest China. Hum. Ecol. Risk Assess. Int. J..

[5] Amini M., Zayeri F., Salehi M. (2021). Trend analysis of cardiovascular disease mortality, incidence, and mortality-to-incidence ratio: Results from global burden of disease study 2017. BMC Public Health.

[6] Lamas G.A., Ujueta F., Navas-Acien A. (2021). Lead and Cadmium as Cardiovascular Risk Factors: The Burden of Proof Has Been Met. J. Am. Heart Assoc..

[7] Sevim Ç., Doğan E., Comakli S. (2020). Cardiovascular disease and toxic metals. Curr. Opin. Toxicol..

[8] Lin C.Y., Lee H.L., Hwang Y.T., Huang P.C., Wang C., Sung F.C., Wu C., Su T.C. (2020). Urinary heavy metals, DNA methylation, and subclinical atherosclerosis. Ecotoxicol. Environ. Saf..

[9] Hong D., Bai Y.P., Gao H.C., Wang X., Li L.F., Zhang G.G., Hu C.P. (2014). Ox-LDL induces endothelial cell apoptosis via the LOX-1-dependent endoplasmic reticulum stress pathway. Atherosclerosis.

[10] Duan H., Zhang Q., Liu J., Li R., Wang D., Peng W., Wu C. (2021). Suppression of apoptosis in vascular endothelial cell, the promising way for natural medicines to treat atherosclerosis. Pharmacol. Res..

[11] Zhang J. (2022). Biomarkers of endothelial activation and dysfunction in cardiovascular diseases. Rev. Cardiovasc. Med..

[12] Lee C.K., Wu C., Lin C.Y., Huang P.C., Sung F.C., Su T.C. (2021). Positive Association between Endothelium-Platelet Microparticles and Urinary Concentration of Lead and Cadmium in Adolescents and Young Adults. Nutrients.

[13] Nguyen T.M.D. (2020). Adiponectin: Role in Physiology and Pathophysiology. Int. J. Prev. Med..

[14] Khoramipour K., Chamari K., Hekmatikar A.A., Ziyaiyan A., Taherkhani S., Elguindy N.M., Bragazzi N.L. (2021). Adiponectin: Structure, Physiological Functions, Role in Diseases, and Effects of Nutrition. Nutrients.

[15] Szydełko J., Trojanowska P., Dąbrowska I., Szydełko-Gorzkowicz M., Litwińczuk M. (2020). Adiponectin as novel biomarker of endothelial dysfunction in insulin resistance and obesity–a narrative review. J. Educ. Health Sport.

[16] Ehsan M., Singh K.K., Lovren F., Pan Y., Quan A., Mantella L.-E., Sandhu P., Teoh H., Al-Omran M., Verma S. (2016). Adiponectin limits monocytic microparticle-induced endothelial activation by modulation of the AMPK, Akt and NFκB signaling pathways. Atherosclerosis.

[17] Nomura S., Inami N., Shouzu A., Omoto S., Kimura Y., Takahashi N., Tanaka A., Urase F., Maeda Y., Ohtani H. (2009). The effects of pitavastatin, eicosapentaenoic acid and combined therapy on platelet-derived microparticles and adiponectin in hyperlipidemic, diabetic patients. Platelets.

[18] Esposito K., Maiorino M.I., Di Palo C., Gicchino M., Petrizzo M., Bellastella G., Saccomanno F., Giugliano D. (2011). Effects of pioglitazone versus metformin on circulating endothelial microparticles and progenitor cells in patients with newly diagnosed type 2 diabetes--a randomized controlled trial. Diabetes Obes. Metab..

[19] Meyer D.N., Crofts E.J., Akemann C., Gurdziel K., Farr R., Baker B.B., Weber D., Baker T.R. (2020). Developmental exposure to Pb(2+) induces transgenerational changes to zebrafish brain transcriptome. Chemosphere.

[20] Kawakami T., Nishiyama K., Kadota Y., Sato M., Inoue M., Suzuki S. (2013). Cadmium modulates adipocyte functions in metallothionein-null mice. Toxicol. Appl. Pharm..

[21] Wu C.-J., Ho A.-C., Chen S.-Y., Pan C.-H., Chuang H.-C., Lai C.-H. (2023). Exposure to Heavy Metals and Serum Adiponectin Levels among Workers: A 2-Year Follow-Up Study. Metabolites.

[22] Ashley-Martin J., Dodds L., Arbuckle T.E., Ettinger A.S., Shapiro G.D., Fisher M., Taback S., Bouchard M.F., Monnier P., Dallaire R. (2015). Maternal blood metal levels and fetal markers of metabolic function. Environ. Res..

[23] Kupsco A., Kioumourtzoglou M.A., Just A.C., Amarasiriwardena C., Estrada-Gutierrez G., Cantoral A., Sanders A.P., Braun J.M., Svensson K., Brennan K.J.M. (2019). Prenatal Metal Concentrations and Childhood Cardiometabolic Risk Using Bayesian Kernel Machine Regression to Assess Mixture and Interaction Effects. Epidemiology.

[24] Valcke M., Ouellet N., Dubé M., Laouan Sidi E.A., LeBlanc A., Normandin L., Balion C., Ayotte P. (2019). Biomarkers of cadmium, lead and mercury exposure in relation with early biomarkers of renal dysfunction and diabetes: Results from a pilot study among aging Canadians. Toxicol. Lett..

[25] Wang X., Karvonen-Gutierrez C.A., Mukherjee B., Herman W.H., Park S.K. (2021). Urinary metals and adipokines in midlife women: The Study of Women’s Health Across the nation (SWAN). Environ. Res..

[26] Lin C.Y., Lee H.L., Hwang Y.T., Wang C., Hsieh C.J., Wu C., Sung F.C., Su T.C. (2020). The association between urine di-(2-ethylhexyl) phthalate metabolites, global DNA methylation, and subclinical atherosclerosis in a young Taiwanese population. Environ. Pollut..

[27] Nie J.M., Li H.F. (2017). Metformin in combination with rosiglitazone contribute to the increased serum adiponectin levels in people with type 2 diabetes mellitus. Exp. Ther. Med..

[28] Chen J.W., Chen H.Y., Li W.F., Liou S.H., Chen C.J., Wu J.H., Wang S.L. (2011). The association between total urinary arsenic concentration and renal dysfunction in a community-based population from central Taiwan. Chemosphere.

[29] Tsai T.L., Kuo C.C., Pan W.H., Chung Y.T., Chen C.Y., Wu T.N., Wang S.L. (2017). The decline in kidney function with chromium exposure is exacerbated with co-exposure to lead and cadmium. Kidney Int..

[30] Landers-Ramos R.Q., Addison O.A., Beamer B., Katzel L.I., Blumenthal J.B., Robinson S., Hagberg J.M., Prior S.J. (2020). Circulating microparticle concentrations across acute and chronic cardiovascular disease conditions. Physiol. Rep..

[31] Pirro M., Schillaci G., Paltriccia R., Bagaglia F., Menecali C., Mannarino M.R., Capanni M., Velardi A., Mannarino E. (2006). Increased ratio of CD31+/CD42- microparticles to endothelial progenitors as a novel marker of atherosclerosis in hypercholesterolemia. Arterioscler. Thromb. Vasc. Biol..

[32] Chiva-Blanch G., Bratseth V., Ritschel V., Andersen G., Halvorsen S., Eritsland J., Arnesen H., Badimon L., Seljeflot I. (2017). Monocyte-derived circulating microparticles (CD14(+), CD14(+)/CD11b(+) and CD14(+)/CD142(+)) are related to long-term prognosis for cardiovascular mortality in STEMI patients. Int. J. Cardiol..

[33] Chirinos J.A., Zambrano J.P., Virani S.S., Jimenez J.J., Jy W., Ahn E., Horstman L.L., Castellanos A., Myerburg R.J., Ahn Y.S. (2005). Correlation between apoptotic endothelial microparticles and serum interleukin-6 and C-reactive protein in healthy men. Am. J. Cardiol..

[34] Sommar J.N., Hedmer M., Lundh T., Nilsson L., Skerfving S., Bergdahl I.A. (2014). Investigation of lead concentrations in whole blood, plasma and urine as biomarkers for biological monitoring of lead exposure. J. Expo. Sci. Environ. Epidemiol..

[35] Kwaifa I.K., Bahari H., Yong Y.K., Noor S.M. (2020). Endothelial Dysfunction in Obesity-Induced Inflammation: Molecular Mechanisms and Clinical Implications. Biomolecules.

[36] Zhou X.-H., Cheng Z.-P., Lu M., Lin W.-Y., Luo L.-L., Ming Z.-Y., Hu Y. (2023). Adiponectin receptor agonist AdipoRon modulates human and mouse platelet function. Acta Pharmacol. Sin..

[37] Deng G., Long Y., Yu Y.R., Li M.R. (2010). Adiponectin directly improves endothelial dysfunction in obese rats through the AMPK–eNOS Pathway. Int. J. Obes..

[38] Cui X.-J., Lin X., Zhong J.-Y., Li S., He J.-Y., Ni Y.-Q., Zhan J.-K., Liu Y.-S. (2020). Adiponectin attenuates the premature senescence of vascular smooth muscle cells induced by high glucose through mTOR signaling pathway. Aging Med..

[39] Malekmohammad K., Bezsonov E.E., Rafieian-Kopaei M. (2021). Role of Lipid Accumulation and Inflammation in Atherosclerosis: Focus on Molecular and Cellular Mechanisms. Front. Cardiovasc. Med..

[40] Patwa J., Flora S.J.S. (2020). Heavy Metal-Induced Cerebral Small Vessel Disease: Insights into Molecular Mechanisms and Possible Reversal Strategies. Int. J. Mol. Sci..

[41] Skoczyńska A., Smolik R. (1994). The effect of combined exposure to lead and cadmium on serum lipids and lipid peroxides level in rats. Int. J. Occup. Med. Environ. Health.

[42] Ahn J., Kim N.S., Lee B.K., Park J., Kim Y. (2018). Association of Blood Pressure with Blood Lead and Cadmium Levels in Korean Adolescents: Analysis of Data from the 2010–2016 Korean National Health and Nutrition Examination Survey. J. Korean Med. Sci..

[43] Lin C.-Y., Hsu S.H.-J., Chen C.-W., Wang C., Sung F.-C., Su T.-C. (2023). Association of Urinary Lead and Cadmium Levels, and Serum Lipids with Subclinical Arteriosclerosis: Evidence from Taiwan. Nutrients.

