# Supplementary material for: The Association among Urinary Lead and Cadmium, Serum Adiponectin, and Serum Apoptotic Microparticles in a Young Taiwanese Population
Source: Nutrients. 2023 Oct 25;15(21):4528. doi: 10.3390/nu15214528 (PMC10647776; doi:10.3390/nu15214528)
Supplement: Supplementary file 1 [file nutrients-15-04528-s001.zip › File S1_section 20230923.pdf]

Supplementary Material

Supplemental methods

Study population and data collection

From 1992 to 2000, approximately 2,615,000 to 2,932,000 school-age children in grades 1 to 12 received an annual urine screening of urine strip by the Chinese Foundation of Health in Taipei, Taiwan. Subjects with abnormal results from two tests for proteinuria, glycosuria, or hematuria underwent a third urine screening test and a general health check-up. A total of 103,756 school children received the health check-ups and the third urine screen. Among these children, 9,227 had elevated blood pressure and 94,529 had normal blood pressure.

From 2006 to 2008 we established a cohort, the YOung TAiwanese Cohort (YOTA) study, based on students with and without childhood EBP, selected from the 1992–2000 urine mass screening population. In the follow-up, we mailed invitation letters to eligible students in the Taipei area. After 3–5 days, 12 trained assistants and nurses conducted telephone interviews inviting those subjects with childhood EBP to come in for a follow-up health examination. No telephone interview contact was made with normotensive students. Among the 707 subjects with EBP in childhood, 303 completed the follow-up health examinations, giving a response rate of 42.9%. Among the 6,390 subjects with normal BP in childhood, 486 completed the follow-up health examinations, giving a response rate of 7.6%. In order to differentiate the effects of environment on age of exposure, we recruited 97

subjects as "best friend controls" in the cohort follow-up period. A total of 886 subjects were included in this study. Physical check-ups were given after written informed consent. All methods in this study were performed in accordance with the relevant guidelines and approved by the Research Ethics Committee of the National Taiwan University Hospital. We excluded 17 individuals with diabetes because their medications could potentially affect adiponectin levels [1]. Another 145 participants were excluded due to a lack of data on urine heavy metals. A total of 724 participants were included in the final analysis.

Measurement of urinary lead and cadmium levels

Collected urine specimens were kept in a freezer (-20°C) until shipment and then immediately stored at −80°C. Samples were analyzed for lead and cadmium following previous published methods [2,3]. After thawing the urine samples, 1 mL of the samples were diluted 10-fold with 9 mL of 1% (v/v) nitric acid (J.T. Baker Chemical Company, Phillipsburg, NJ, USA) in 15 mL polypropylene tubes and analyzed by Inductively coupled plasma-mass spectrometry (ICP-MS, 7700 series; Agilent Technologies, Inc., Santa Clara, CA, USA).

A guideline of quality control and method detection limit (MDL) were modified from the National Institute of Environmental Analysis PA-103, PA-104, and PA-107, Taiwan EPA (NIEA PA-103, PA-104, PA-107, 2005) [4], which is based on the standards of the National Environmental Laboratory Accreditation [5]. The calibration curves ranged from 0.01 to 50 μg/L for lead and cadmium (SPEX CertiPrep; Multi-element

Solution 2, CLMS-2, Metuchen, NJ, USA) with correlation coefficient [R^2] >0.995. To ensure the quality of analysis results, one sample of blank, spiked, duplicate, and quality control was tested in every batch of 10 samples. The concentration of lead and cadmium in the blank sample was less than two-fold of the MDL, while the recoveries of 1 μg/L spiked urine and 5 μg/L quality control samples were within ±20%, Each sample was run in duplicate within ±10% precision. If lead in urine sample was below MDL (Lead : 0.007 μg/L, Cadmium: 0.006 μg/L), it would be replaced by one-half the MDL.

Covariates

During the interview, demographic data were gathered. Household income was classified as either above or below 50,000 New Taiwan Dollars (NTD) per month. Alcohol consumption was categorized as current consumption or non-consumption. Smoking status was differentiated into non-active smokers and active smokers. The body mass index (BMI) was calculated as the weight in kilograms divided by the square of the height in meters. For participants aged ≥20 years, the BMI z-score was determined using the equation (BMI of each participant - mean of BMI) / (standard deviation of BMI), while for those aged 12-19 years, it was computed using the WHO anthropometric calculator [6]. Blood pressure was measured twice after a 3-minute rest period, utilizing a mercury manometer. In adults, hypertension was defined as self-reported current use of antihypertensive medication or an average systolic blood

pressure ≥140 mmHg or average diastolic blood pressure ≥90 mmHg. Childhood hypertension was determined based on blood pressure values meeting or exceeding the modified sex- and age-specific criteria [7].

Supplemental Table S1. Basic demographics of the subjects, including the geometric mean (geometric SD) of urinary Pb and Cd concentrations.

	N (%)	Pb (μg/g creatinine)		Cd (μg/g creatinine)	
		Mean (SD)	*P* value	Mean (SD)	*P* value
Total	724 (100)	1.50 (5.36)		0.63 (5.36)	
Sex			<0.001		<0.001
Female	434 (59.9)	1.83 (5.33)		0.75 (3.41)	
Male	290 (40.1)	1.12 (5.18)		0.63 (3.61)	
Age (years)			<0.001		<0.001
12–19	235 (31.9)	2.25 (6.39)		0.84 (3.78)	
20–30	503 (68.1)	1.25 (4.75)		0.56 (3.38)	
Household income			0.796		0.852
< 50000 TWD	283 (39.1)	1.54 (5.43)		0.64 (3.61)	
≥ 50000 TWD	441 (60.9)	1.49 (5.34)		0.63 (3.52)	
Smoking status			0.144		0.011
Not active	350 (47.4)	1.62 (5.64)		0.67 (3.58)	
Active smoker	123 (16.7)	1.15 (4.81)		0.46 (3.41)	
Current alcohol consumption			0.894		0.803
No	668 (90.6)	1.50 (5.38)		0.64 (3.56)	
Yes	69 (9.4)	1.55 (5.26)		0.61 (3.54)	
Hypertension			0.030		0.052
No	678 (91.9)	1.45 (5.14)		0.62 (3.47)	
Yes	60 (8.1)	2.36 (7.86)		0.86 (4.35)	

P values determined by *t*-test or ANOVA

Supplemental Table S2: Geometric mean (S.E.) of apoptotic microparticles in different Pb and Cd subgroups in the linear regression model.

		Pb ≤ 50th and Cd ≤ 50th	Pb > 50th and Cd ≤ 50th	Pb ≤ 50th and Cd >50	Pb > 50th and Cd > 50th
CD31+/CD42a−	Geometric mean (S.E.)	14.27 (1.24)	13.60 (1.73)	14.70 (1.76)	16.85 (1.21)
	P value	Reference	0.644	0.772	0.002
	P for trend	0.008			
CD31+/CD42a+	Geometric mean (S.E.)	2291.59 (1.14)	4142.27 (1.27)	3118.17 (1.27)	6542.01 (1.14)
	P value	Reference	0.016	0.205	<0.001
	P for trend	<0.001			
CD14	Geometric mean (S.E.)	106.80 (1.04)	109.29 (1.08)	115.12 (1.08)	126.84 (1.04)
	P value	Reference	0.763	0.316	<0.001
	P for trend	0.001			

Model adjusted for age, gender, BMI z score, smoking status, drinking status, household income

Abbreviations: Cd: cadmium; Pb: lead; BMI: body mass index

References

1. Nie, J.M.; Li, H.F. Metformin in combination with rosiglitazone contribute to the increased serum adiponectin levels in people with type 2 diabetes mellitus. *Exp Ther Med* **2017**, *14*, 2521-2526, doi:10.3892/etm.2017.4823.
2. Chen, J.W.; Chen, H.Y.; Li, W.F.; Liou, S.H.; Chen, C.J.; Wu, J.H.; Wang, S.L. The association between total urinary arsenic concentration and renal dysfunction in a community-based population from central Taiwan. *Chemosphere* **2011**, *84*, 17-24, doi:10.1016/j.chemosphere.2011.02.091.
3. Tsai, T.L.; Kuo, C.C.; Pan, W.H.; Chung, Y.T.; Chen, C.Y.; Wu, T.N.; Wang, S.L. The decline in kidney function with chromium exposure is exacerbated with co-exposure to lead and cadmium. *Kidney Int* **2017**, *92*, 710-720, doi:10.1016/j.kint.2017.03.013.
4. Liao, K.-W.; Pan, W.-H.; Liou, S.-H.; Sun, C.-W.; Huang, P.-C.; Wang, S.-L. Levels and temporal variations of urinary lead, cadmium, cobalt, and copper exposure in the general population of Taiwan. *Environmental Science and Pollution Research* **2019**, *26*, 6048-6064.
5. NELAC. NELAC Standard. Availabe online: https://nelac-institute.org/docs/2003nelacstandard.pdf (accessed on July 30).
6. WHO. Global Database on Child Growth and Malnutrition. Availabe online: http://www.who.int/nutgrowthdb/software/en/ (accessed on March 15).
7. Battistoni, A.; Canichella, F.; Pignatelli, G.; Ferrucci, A.; Tocci, G.; Volpe, M. Hypertension in Young People: Epidemiology, Diagnostic Assessment and Therapeutic Approach. *High blood pressure & cardiovascular prevention : the official journal of the Italian Society of Hypertension* **2015**, *22*, 381-388, doi:10.1007/s40292-015-0114-3.
